# Supplementary material for: Interpreting random forest analysis of ecological models to move from prediction to explanation
Source: Sci Rep. 2023 Mar 8;13:3881. doi: 10.1038/s41598-023-30313-8 (PMC9995331; doi:10.1038/s41598-023-30313-8)
Supplement: Supplementary file 1 — Supplementary Information 1. [file 41598_2023_30313_MOESM1_ESM.zip › SF1-CompadreAnalysisRMD.html]

Compadre Analysis


# Compadre Analysis

#### Paul Glaum as part of Simon et al 2022

#### 2022-04-08

# Compadre plant matrix repository

COMPADRE is a global online repository for matrix population models
(MPMs) and metadata on plants Please see https://jonesor.github.io/Rcompadre/index.html & https://jonesor.github.io/CompadreGuides/user-guide.html
for in-depth discussion of COMPADRE install and usage.

## Install the stable release package from CRAN. Also load ggplot:

```
#install.packages("Rcompadre")
##Remote install from github with:
#remotes::install_github("jonesor/Rcompadre", build_vignettes = FALSE)
##
library(Rcompadre)
require(ggplot2)
require(gridExtra)
library(kableExtra)
library(leaflet)
```

## Get up to date version of COMPADRE

```
compadre <- cdb_fetch("compadre")
```

```
## This is COMPADRE version 6.22.5.0 (release date May_11_2022)
## See user agreement at https://compadre-db.org/Help/UserAgreement
## See how to cite at https://compadre-db.org/Help/HowToCite
```

### Stage No. distribution

Check the current distribution of different matrix structures in
COMPADRE. We only show models made up of 10 stages or less:

```
## 
##    1    2    3    4    5    6    7    8    9   10 
##    4  518 1251 2121 1785 1551  472  375  202  175
```

## Check the breakdown of 3-stage plant models.

### Species

Check number of taxonomic species currently represented by 3-stage
plant models:

```
## [1] 110
```

### Genera

Check distribution of taxonomic genera currently represented by 3-stage
plant models:

Breakdown across the 89 plant genera

| Plant Genus | Freq in Database |
| --- | --- |
| Acer | 10 |
| Achillea | 2 |
| Actinostemon | 2 |
| Adenocarpus | 17 |
| Adesmia | 2 |
| Aechmea | 36 |
| Alliaria | 50 |
| Allium | 1 |
| Amaranthus | 12 |
| Anarrhinum | 6 |
| Antirrhinum | 13 |
| Arctophila | 3 |
| Arenaria | 6 |
| Aristida | 8 |
| Aster | 27 |
| Astragalus | 5 |
| Boltonia | 15 |
| Brassica | 41 |
| Caladenia | 9 |
| Calochortus | 4 |
| Carduus | 2 |
| Carlina | 9 |
| Cedrela | 1 |
| Centaurea | 178 |
| Chaerophyllum | 3 |
| Cheirolophus | 6 |
| Cirsium | 40 |
| Clarkia | 21 |
| Clintonia | 5 |
| Commelina | 4 |
| Conradina | 3 |
| Corallorhiza | 6 |
| Cornus | 1 |
| Cucurbita | 9 |
| Digitaria | 8 |
| Echinochloa | 12 |
| Eryngium | 3 |
| Euphorbia | 6 |
| Fabiana | 4 |
| Fragaria | 111 |
| Fritillaria | 2 |
| Helianthemum | 6 |
| Heliconia | 24 |
| Iris | 1 |
| Jacobaea | 96 |
| Jurinea | 6 |
| Koeleria | 2 |
| Kosteletzkya | 9 |
| Kummerowia | 2 |
| Kunkeliella | 6 |
| Laserpitium | 6 |
| Lepidium | 39 |
| Limonium | 6 |
| Lindera | 1 |
| Linum | 26 |
| Mimulus | 29 |
| Mulinum | 2 |
| Murdannia | 4 |
| Narcissus | 3 |
| Neotinea | 6 |
| Oenothera | 9 |
| Parolinia | 6 |
| Pediocactus | 110 |
| Pinguicula | 27 |
| Plantago | 3 |
| Platanthera | 4 |
| Potentilla | 1 |
| Primula | 2 |
| Ptychosperma | 1 |
| Ranunculus | 7 |
| Raphanus | 27 |
| Salix | 6 |
| Sarcocapnos | 10 |
| Scabiosa | 1 |
| Sclerocarya | 1 |
| Scorzonera | 1 |
| Senecio | 2 |
| Sequoia | 1 |
| Spartina | 1 |
| Sporobolus | 6 |
| Stipa | 6 |
| Taraxacum | 2 |
| Telipogon | 1 |
| Tetraneuris | 3 |
| Thymus | 7 |
| Trifolium | 2 |
| Vella | 12 |
| Verbascum | 12 |
| Vitaliana | 3 |

### Families

Check distribution of taxonomic families currently represented by
3-stage plant models:

Breakdown across the 46 plant families

| Plant Family | Freq in Database |
| --- | --- |
| Amaranthaceae | 12 |
| Amaryllidaceae | 4 |
| Anacardiaceae | 1 |
| Apiaceae | 14 |
| Arecaceae | 1 |
| Asteraceae | 274 |
| Brassicaceae | 175 |
| Bromeliaceae | 36 |
| Cactaceae | 110 |
| Caprifoliaceae | 1 |
| Caryophyllaceae | 6 |
| Cistaceae | 6 |
| Commelinaceae | 8 |
| Compositae | 115 |
| Cornaceae | 1 |
| Cucurbitaceae | 9 |
| Cupressaceae | 1 |
| Euphorbiaceae | 8 |
| Fabaceae | 5 |
| Heliconiaceae | 24 |
| Iridaceae | 1 |
| Lamiaceae | 10 |
| Lauraceae | 1 |
| Leguminosae | 21 |
| Legumiosae | 2 |
| Lentibulariaceae | 27 |
| Liliaceae | 11 |
| Linaceae | 26 |
| Malvaceae | 9 |
| Meliaceae | 1 |
| Onagraceae | 30 |
| Orchidaceae | 26 |
| Papaveraceae | 10 |
| Phrymaceae | 26 |
| Plantaginaceae | 22 |
| Plumbaginaceae | 6 |
| Poaceae | 46 |
| Primulaceae | 5 |
| Ranunculaceae | 7 |
| Rosaceae | 112 |
| Salicaceae | 6 |
| Santalaceae | 6 |
| Sapindaceae | 10 |
| Scrophulariaceae | 13 |
| Scropulariaceae | 2 |
| Solanaceae | 4 |

### Geographic distribution:

Check COMPADRE website above to see Eco-region abbreviations.

Breakdown across continents

| Continent | Freq in Database |
| --- | --- |
| Africa | 17 |
| Asia | 2 |
| Europe | 604 |
| N America | 534 |
| Oceania | 16 |
| S America | 74 |

Breakdown across Regions

| Eco-Region | Freq in Database |
| --- | --- |
| BOR | 36 |
| DES | 141 |
| FGS | 4 |
| MED | 376 |
| MON | 2 |
| TBM | 330 |
| TCF | 78 |
| TGS | 93 |
| TGV | 10 |
| TMB | 65 |
| TUN | 6 |

#### Map

```
##Create a subset of only 3-stage matrices
mat3=subset(compadre, MatrixDimension==3);
LLdf=data.frame(mat3$Lat,mat3$Lon,mat3$fullSpName);
colnames(LLdf)=c("lat","long","Species");
LLdf<- LLdf[complete.cases(LLdf), ];
LLdf<-LLdf[!duplicated(LLdf), ];

suppressWarnings(
  LLdf %>%
  leaflet( width = 900) %>%
  addTiles() %>%
  addMarkers(clusterOptions = markerClusterOptions(), popup = ~paste0(
      "<b><h4>","Species", "</h4></b><br>",LLdf$Species)  )
)
```

## Prepare demographic rates

Pull specific demographic rate data from the 3-stage models. Note, if
you are running these script, this must be run before making figures
below.

```
##Create a subset of only 3-stage matrices
mat3=subset(compadre, MatrixDimension==3);
##COMPADRE uses "accessor functions" to obtain specific parts of the matrices.
##matF accesses the fecundity part of the matrix, akin to our rF
Fmats=matF(mat3);
##matU accesses the survival-related transitions, akin to our g12 and g2F
Umats=matU(mat3);

#To store entry. Fs for reproduction alone, F.U for reproduction & transitions. 
Fs=rep(); F.U=matrix(0,length(Fmats),4)
for(i in 1:length(Fmats) ) {
    Fs[i]=sum(Fmats[i][[1]][1,3] )
    F.U[i,1:3]=Umats[i][[1]][lower.tri(Umats[i][[1]])] ##only take the transitions
    F.U[i,4]=Fs[i] #include reproduction
}
#make easy to use data frame for our curated data. 
#name columns in the vein of our parameters. Note, d31 named for the 
#addition into stage 1 from stage 3 is the same as our rF
UnF=as.data.frame(F.U)
colnames(UnF)=c('g12','g13','g23','d31')
UnF=subset(UnF,UnF$d31>0&UnF$d31<100)
```

## Make plots

Scatter plots showing correlations between parameters:

```
g12vrF=ggplot(UnF) + theme_bw() +
 geom_point(aes(x=g12,y=log(d31)),alpha=.4,size=2) +
 xlab(bquote('maturation rate'~(g['12'])) )+
 ylab(bquote('log(reproduction rate)'~(r[F])) )

g23vrF=ggplot(UnF) + theme_bw() +
 geom_point(aes(x=g23,y=log(d31)),alpha=.4,size=2) +
 xlab(bquote('maturation rate'~(g['2F'])) )+
 ylab(bquote('log(reproduction rate)'~(r[F])) )

g23vg12=ggplot(UnF) + theme_bw() +
 geom_point(aes(x=g23,y=g12),alpha=.4,size=2) +
 xlab(bquote('maturation rate'~(g['2F'])) )+
 ylab(bquote('germination rate'~(g['12'])) )

suppressWarnings(grid.arrange(g12vrF, g23vrF, g23vg12, ncol=3) )
```

Histograms of the value distribution for each parameter:

```
Fss=Fs[Fs<100&Fs>0]

h1<-suppressWarnings(hist(na.omit(Fss),breaks=80,col='light blue',xlim=c(0,10.6),plot=FALSE));
h1$density = suppressWarnings(h1$counts/sum(h1$counts));
h2<-suppressWarnings(hist(na.omit(UnF$g12[UnF$g12>0]),breaks=40,col='light blue',plot=FALSE));
h2$density = suppressWarnings(h2$counts/sum(h2$counts));
h3<-suppressWarnings(hist(na.omit(UnF$g23[UnF$g23>0]),breaks=40,col='light blue',plot=FALSE));
h3$density = suppressWarnings(h3$counts/sum(h3$counts))

par( mfrow= c(1,3) )
plot(h1,freq=FALSE,col='light blue',xlim=c(0,10.6),
xlab="Measured Rate",ylab="Density",main="Reproduction into 1st Stage")#,ylim=c(0,.5))

plot(h2,freq=FALSE,col='light blue',
xlab="Measured Rate",ylab="Density",main="1st to 2nd Stage Transition")#,ylim=c(0,.5))

plot(h3,freq=FALSE,col='light blue',
xlab="Measured Rate",ylab="Density",main="2nd to 3rd Stage Transition")#,ylim=c(0,.5))
```
