# Supplementary material for: Interpreting random forest analysis of ecological models to move from prediction to explanation
Source: Sci Rep. 2023 Mar 8;13:3881. doi: 10.1038/s41598-023-30313-8 (PMC9995331; doi:10.1038/s41598-023-30313-8)
Supplement: Supplementary file 1 — Supplementary Information 1. [file 41598_2023_30313_MOESM1_ESM.zip › SF2-randomForestCode.html]

Random Forest & Linear Functions


# Random Forest & Linear Functions

#### Paul Glaum as part of Simon et al 2022

#### 2022-05-17

### Load packages

```
library(readxl)
library(readr)
library(dplyr)
library(tidymv)
library(ggplot2)

##These packages need to be loaded for analysis:
library(randomForest) #for random forests
library(pROC) #for estimating AUC 
#library(Metrics) #for evaluating rsme, loading this messes up the auc function for
            #pROC...
library(pls) #for partial least squares regression

library(iml)
library("future")
library("future.callr")
require(gridExtra)
library(kableExtra)
```

This is an R Markdown document. Markdown is a simple formatting
syntax for authoring HTML, PDF, and MS Word documents. For more details
on using R Markdown see http://rmarkdown.rstudio.com.

### Load data & create necessary functions

```
setwd("C:/Users/prglaum/Documents/Stage-Structure/SimonEtAl2021/NEWESTFiveParamSweep-alp0.1-hF1-h21")
file.list <- list.files(pattern='*.csv')
df.list <- lapply(file.list, read_csv)
```

```
## Warning: One or more parsing issues, see `problems()` for details
## One or more parsing issues, see `problems()` for details
```

```
h1a1 <- bind_rows(df.list, .id = "id")
##need to run these too
h1a1$rF=round(h1a1$rF,1)
h1a1$g1=round(h1a1$g1,2)
h1a1$g2=round(h1a1$g2,2)
h1a1$a2=round(h1a1$a2,1)
h1a1$aF=round(h1a1$aF,1)

#The following equations form the output from our model's subfunctions
#delta, gamma12(gam1), gamma2F(gam2), thetaF and thetaS2 in order to 
#make L:D ratio
h1a1$delta=h1a1$Feqm*(h1a1$rF - h1a1$alpF*h1a1$Feqm)
h1a1$gam1=( h1a1$g1*h1a1$S1eqm/(1 + h1a1$alp1*(h1a1$Feqm + .2*(h1a1$S1eqm + h1a1$S2eqm))) )
h1a1$gam2=( h1a1$g2*h1a1$S2eqm/(1 + h1a1$alp2*(h1a1$Feqm + .2*(h1a1$S2eqm))) )
h1a1$thetaF= ((h1a1$Heqm*.6*h1a1$aF*h1a1$Feqm)/(1+h1a1$aF*h1a1$hF*h1a1$Feqm + h1a1$a2*h1a1$h2*h1a1$S2eqm) )
h1a1$thetaS2= ((h1a1$Heqm*.6*h1a1$a2*h1a1$S2eqm)/(1+h1a1$aF*h1a1$hF*h1a1$Feqm + h1a1$a2*h1a1$h2*h1a1$S2eqm) )
h1a1$thetas=h1a1$thetaF+h1a1$thetaS2
h1a1$LDratio=((h1a1$thetaF/.6)+(h1a1$thetaS2/.6))/(h1a1$S1eqm+h1a1$S2eqm+h1a1$Feqm)
```

For the sake of expendiency, we only focus on a herbivory subset of
the full simulation data set. Given the size of our dataset, running the
RF on the full simulation data set does have a prolonged running time.
We name the temporary data set, “temp.”

```
temp=subset(h1a1,aF==1&a2==0.0)
temp=data.frame(temp$StableB,temp$MaxEVal,temp$rF,temp$g1,temp$g2,temp$LDratio,temp$gam1,temp$gam2);
colnames(temp)=c('Stable','EV','rF','g1','g2','LDratio','gam1','gam2'); temp$Stable=as.factor(temp$Stable);
```

## Cross Validation set up

We will split the data into training and validation subsets. The
training data is ~2/3 of the available data and is used to train the RF
on our predictors and dependent variables. The validation data makes up
the remaining 1/3 of the available data and tests the performance of the
model on data not used to train the model.

```
data_set_size <- floor(nrow(temp)/1.5)
# Generate a random sample of "data_set_size" indexes
indexes <- sample(1:nrow(temp), size = data_set_size)
# Assign the data to the correct sets
training <- temp[indexes,]
validation1 <- temp[-indexes,]
```

## Random Forest: CATEGORIZATION Task

Analysis using categories (stable and unstable) ### Train RF on
training set

Random Forest Parameters: mtry = 2, ntrees = 500, number of trees to
produce.

```
rfCat=randomForest(as.factor(Stable) ~ rF+g1+g2, data=training, ntree=500,mtry=2,keep.forest=TRUE,importance=TRUE)
```

Check the variable importance in random forest predictions on
training set.

```
params=as.vector(names((rfCat$importance[,3]*100)))
imps=as.vector(as.numeric(rfCat$importance[,3]*100))
DF=data.frame(params,imps); #DF$imps=as.numeric(as.character(DF$imps));
ggplot(DF, aes(x=params,y=imps))+ #,fill=imps
   theme(axis.text = element_text(size = 14)) +
   geom_bar(stat="identity", position="dodge")+coord_flip()+
   ylab("Normalized Variable Importance")+
   xlab("Feature/Predictor")+
   ggtitle("Information Value Summary")+
   scale_x_discrete(breaks = c("rF", "g1", "g2"),
                   labels = c(expression( r["F"]), expression( g[12]), expression( g["2F"])) )
```

## Run random forest (rfCat) on validation data

```
catPred=predict(rfCat,newdata=validation1[,3:5],type=c("response"))
```

### Check the accuracy

Confusion matrix

```
table(observed=validation1[,1],predicted=catPred)
```

```
##         predicted
## observed    0    1
##        0  634    8
##        1   14 2278
```

ROC curve & AUC measurement

```
validation1$pred=catPred
g <- roc(validation1$Stable ~ as.numeric(as.character(catPred)))#, data = validation1)
gAUC=g$auc;
plot(g,main=paste("AUC =",gAUC))
```

### Feature effects on prediction

This uses the iml package

```
#rfCat=randomForest((Stable) ~ rF+g1+g2, data=training, ntree=400,mtry=2,keep.forest=TRUE,importance=TRUE)
predictor <- Predictor$new(rfCat, data = validation1[,3:5], y = validation1[,1],class = 2)
future::plan("callr", workers = 4)
pdp2F=FeatureEffect$new(predictor, feature = c("g1","g2"),method="pdp")
#the plot feature of the PDP uses ggplot so we can modify the figure output
pdp2F$plot()+theme_bw()+
  theme(text = element_text(size=14)) +
  xlab(bquote('Base Germination Rate '~(g[12])~'') ) +
  ylab(bquote('Base Seedling Maturation Rate '~(g["2F"])~'') ) +
  scale_fill_gradient(name=bquote('Est.\nstability\nprobability '))
```

### Predict with linear model using factors

GLM for CATEGORICAL predictions using our ecological “factors”

```
###GLM for CATEGORICAL PREDICTIONS###
gMod=glm(as.factor(Stable)~LDratio+gam1+gam2,data=training,family='binomial')
```

```
## Warning: glm.fit: fitted probabilities numerically 0 or 1 occurred
```

```
probPred=predict(gMod,validation1[,6:8],type=c("response"))
validation1$prob=probPred
g <- roc(Stable ~ prob, data = validation1)
gAUC=g$auc;
plot(g, main=paste("AUC =",gAUC))
```

## Random Forest: REGRESSION Task

Analysis using continuous variable:maximum eigenvalue ### Train RF on
training set

Random Forest Paramters mtry = 2, ntrees = 500

```
rfReg=randomForest(EV ~ rF+g1+g2, data=training, ntree=500,mtry=2,keep.forest=TRUE,importance=TRUE)
```

Check the variable importance in random forest predictions on
training set.

```
params=as.vector(names((rfReg$importance[,2]*100)))
imps=as.vector(as.numeric(rfReg$importance[,2]*100))
DF=data.frame(params,imps); #DF$imps=as.numeric(as.character(DF$imps));
ggplot(DF, aes(x=params,y=imps))+ #,fill=imps
   theme(axis.text = element_text(size = 14)) +
   geom_bar(stat="identity", position="dodge")+coord_flip()+
   ylab("Normalized Variable Importance")+
   xlab("Feature/Predictor")+
   ggtitle("Information Value Summary")+
   scale_x_discrete(breaks = c("rF", "g1", "g2"),
                   labels = c(expression( r["F"]), expression( g[12]), expression( g["2F"])) )
```

### Feature effects on prediction

This uses the iml package

```
predictor <- Predictor$new(rfReg, data = validation1[,3:5], y = validation1[,1],class = 2)
future::plan("callr", workers = 4)
pdp2F=FeatureEffect$new(predictor, feature = c("g1","g2"),method="pdp")
#the plot feature of the PDP uses ggplot so we can modify the figure output
pdp2F$plot()+theme_bw()+
  theme(text = element_text(size=14)) +
  xlab(bquote('Base Germination Rate '~(g[12])~'') ) +
  ylab(bquote('Base Seedling Maturation Rate '~(g["2F"])~'') ) +
  scale_fill_gradient(name=bquote('Est. max\neigenvalue '))
```

### Predict with linear model using factors

Partial Least Squares for REGRESSION predictions using our ecological
“factors”

```
tempPLSmod=plsr(EV~LDratio+gam1+gam2,data=training)
plsPred=predict(tempPLSmod,newdata=validation1[,6:8])
```

### Compare accuracy

```
library(Metrics) #for evaluating rsme, loading this messes up the auc function for pROC...

regPredRF=predict(rfReg,newdata=validation1[,3:5],type=c("response"))
rmseRF=rmse(validation1$EV,regPredRF)
rmsePLS=rmse(validation1$EV,plsPred)

qw=data.frame(c(rmseRF,rmsePLS));
colnames(qw)="RMSE";
rownames(qw)=c("Random Forest: simulation parameters","Partial Least Squares: ecological factors")

knitr::kable(qw,caption = "Comparing RMSE between random forest on simulation model parameters and partial least squares on ecological factors", floating.environment="sidewaystable") %>% 
  kable_styling("striped", full_width = F)
```

Comparing RMSE between random forest on simulation model parameters and
partial least squares on ecological factors

|  | RMSE |
| --- | --- |
| Random Forest: simulation parameters | 0.0001836 |
| Partial Least Squares: ecological factors | 0.0028210 |
